# Left hypoplastic lung and hemoptysis—rare familial unilateral pulmonary vein atresia

**DOI:** 10.1002/ccr3.2982

**Published:** 2020-06-08

**Authors:** Ronly Har‐Even Cohn, Matthew Hicks, Atilano Lacson, Anne Hicks

**Affiliations:** ^1^ Department of Pediatrics University of Alberta Edmonton AB Canada; ^2^ Department of Laboratory Medicine and Pathology University of Alberta Edmonton AB Canada

**Keywords:** pulmonary hypertension (PH), pulmonary vein stenosis (PVS), unilateral pulmonary vein atresia (UPVA)

## Abstract

Unilateral pulmonary vein atresia (UPVA) is a rare congenital vascular malformation with obliteration of the pulmonary vein. We present a case series of three siblings with variable presentation of UPVA. We suggest a dominant genetic cause based on different paternity. Identifying genetic etiology would contribute to early diagnosis and screening.

## Graphical abstract

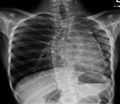

## 1 INTRODUCTION

Unilateral pulmonary vein atresia (UPVA) is a rare vascular abnormality associated with significant morbidity and mortality. UPVA is a progressive and severe form of pulmonary vein stenosis (PVS). We present a familial case series showing varying presentation of left (L) PVA in 3 siblings.

## 2 CASE 1

**Figure 1:**
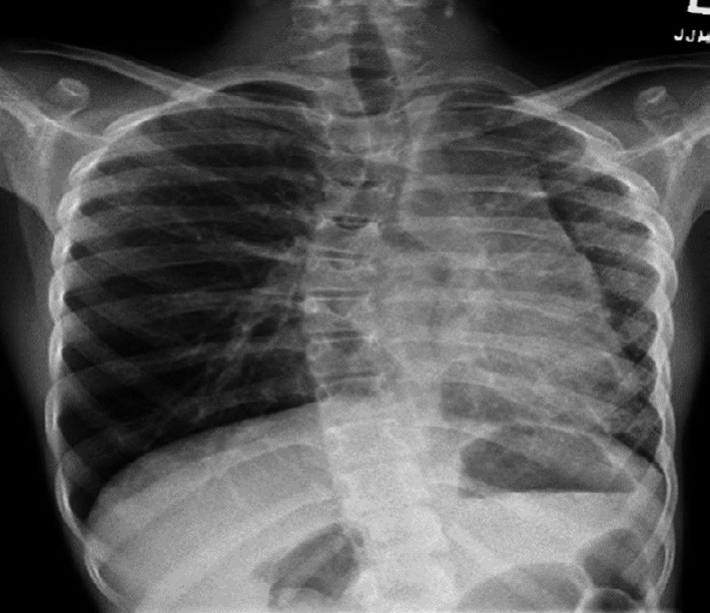
CXR—L hypoplasia

The index case was an 11‐year‐old female immigrant from Africa with a history of first‐degree AV block, scoliosis, and previous exposure to TB (Mantoux positive, interferon gamma negative). Her respiratory symptoms started at 10 year of age with transient cough and chest pain. Her physical examination showed decreased air entry to the left (L) side. On chest X‐ray (CXR), she had L volume loss with mediastinal shift and hyperinflation of the right (R) lung as shown in Figure 1. Echocardiography demonstrated small L pulmonary artery (PA); however, her L PV was not seen. There were no signs of pulmonary hypertension (PH). At that time, she was treated conservatively. A year later, she developed persistent hemoptysis.

**Figure 2:**
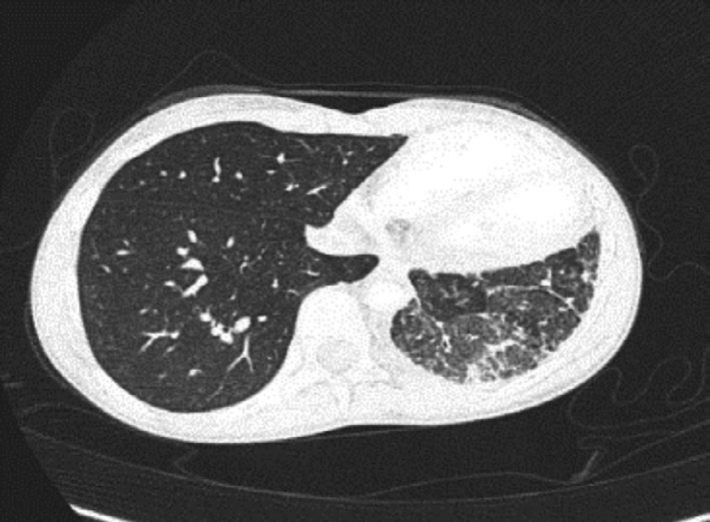
CTA—abnormalities in lung parenchyma and vasculature. The pulmonary veins were not demonstrated near the LA

On admission, her vital signs were normal other than fever**.** She had decreased air entry to the L base with no increased respiratory effort or clubbing. Laboratory investigations (CBC, coagulation factors, blood gases, ferritin, and sputum culture) were normal. Chest computed tomography angiography (CTA) revealed L pulmonary hypoplasia with diffuse parenchymal abnormalities including patchy ground glass opacities and interlobular septal thickening attributed to pulmonary hemorrhage, a small L PA, and tiny mediastinal collateral vessels. The L PVs were not seen near the L atrium (LA). CTA is shown in Figure 2.

**Figure 3:**
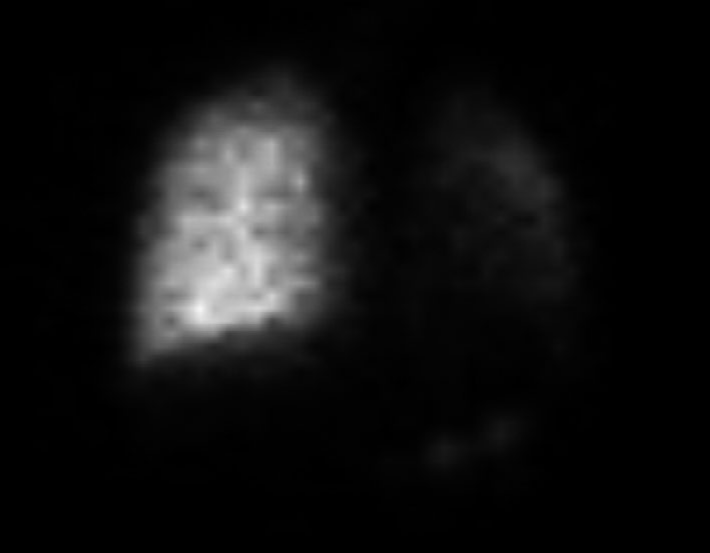
V/Q scan—severe decreased L ventilation and perfusion

During her admission, hemoptysis progressed. Urgent bronchoscopy revealed diffuse bleeding from the lower division of the L lower bronchus. It was treated with 1% xylocaine and adrenaline with no improvement. A V/Q scan showed decreased perfusion and ventilation of the L lung (6% and 19%, respectively) as shown in Figure 3.

Due to persistent hemoptysis and minimal perfusion, she had a L pneumonectomy.

**Figure 4:**
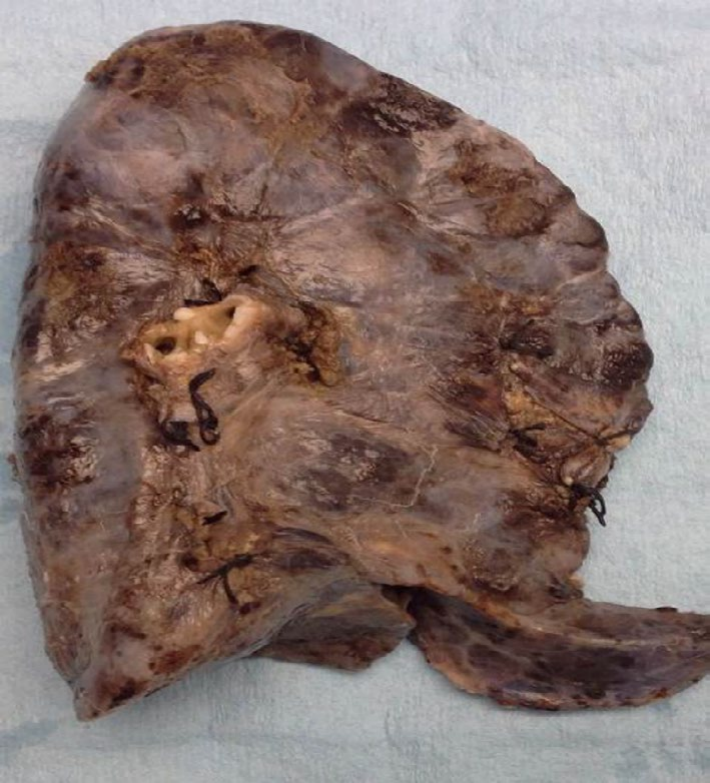
Gross photograph of left lung with focal pleural fibrosis and consolidation of the upper lob. Encircled, ligated arterial collaterals

**Figure 5:**
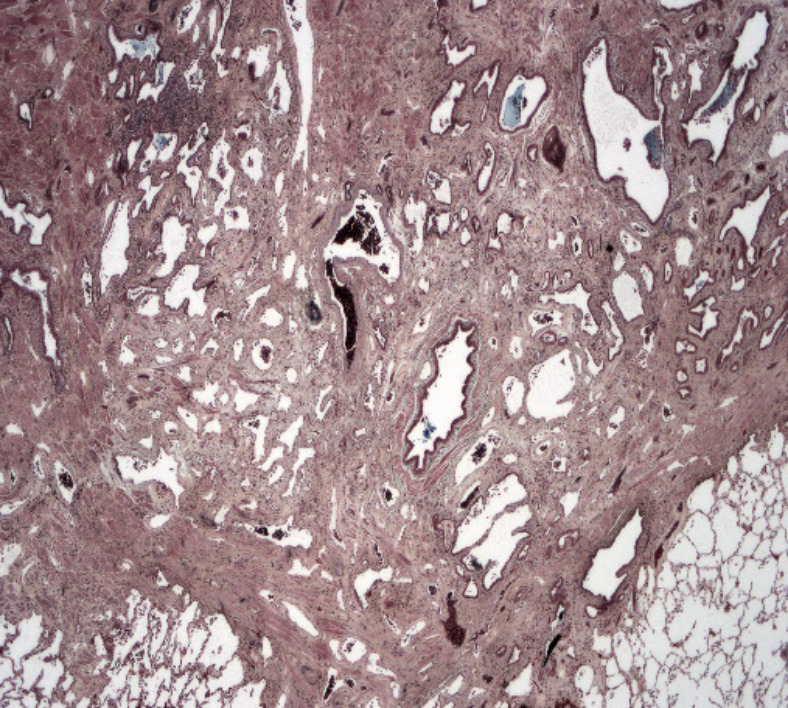
A low‐power view of left upper lobe stained with MOVAT to show consolidation with honeycombed fibrosis

Histopathology showed an extremely fibrotic L lung. The L PV was diminished in size. The lung had a normal weight with normal alveolar structures. Dilated pleural collaterals resembling pulmonary arteriovenous malformation (PAVM) were noted by the surgeon. Pathology showed a left lower lobe parenchymal hemorrhage, left upper lobe focal consolidation with fibrosis, and focal segmental cystic lymphangiectasia compatible with a diagnosis of unilateral pulmonary vein atresia (UPVA). The cause of bleeding was attributed to venous infarction or rupture of collateral circulation or abnormal anastomoses. Gross pathology picture is shown in Figure 4. Histopathology slide is shown in Figure 5.

Since her L pneumonectomy, the patient is doing well despite scoliosis, restrictive lung function (VC 45%, TLC 47%), and intermittent chest pain. She is active in sports with minimal exercise intolerance.

## 3 CASE 2

Her younger half‐brother was born preterm at 26‐week gestation by urgent cesarean section for fetal distress. Pregnancy was complicated by gestational hypertension and pregnancy‐induced cholestasis. Birth weight was 715 g. Apgar scores were 7, 9, and shortly after birth, he was intubated and given surfactant for respiratory distress. His course was complicated by prolonged intubation with high‐frequency ventilation and pulmonary hemorrhage. Other complications included bronchopulmonary dysplasia (BPD), patent ductus arteriosus (PDA) (ligated), necrotizing enterocolitis (NEC) (ileostomy), vesicoureteral reflux (VUR) grades 2‐3 (multiple urinary tract infections on amoxicillin prophylaxis), congenital glaucoma, parenteral nutrition‐related conjugated hyperbilirubinemia (resolved), bilateral inguinal hernia (repaired), osteopenia of prematurity, apnea of prematurity (resolved), frequent episodes of sepsis (resolved), and known bacterial airway colonization with *Staphylococcus aureus* and *Stenotrophomonas maltophilia*. He weaned from oxygen support at 8 months of age.

**Figure 6:**
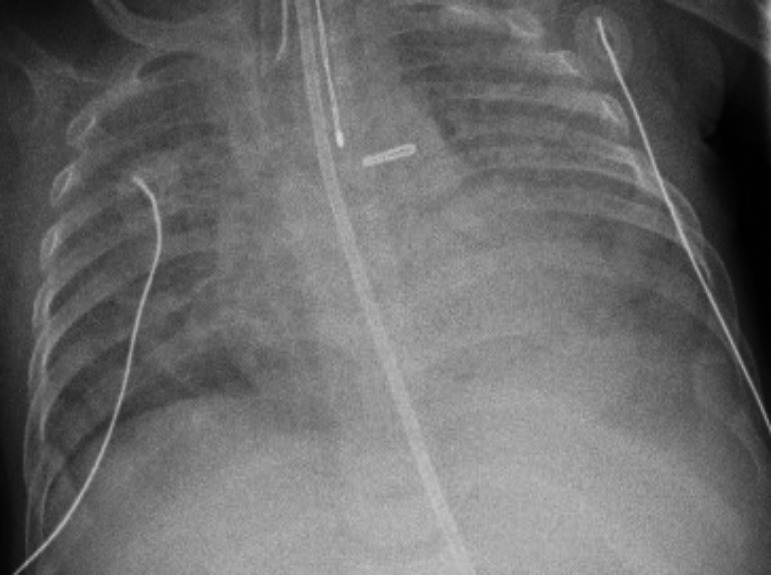
CXR—bilateral opacities

**Figure 7:**
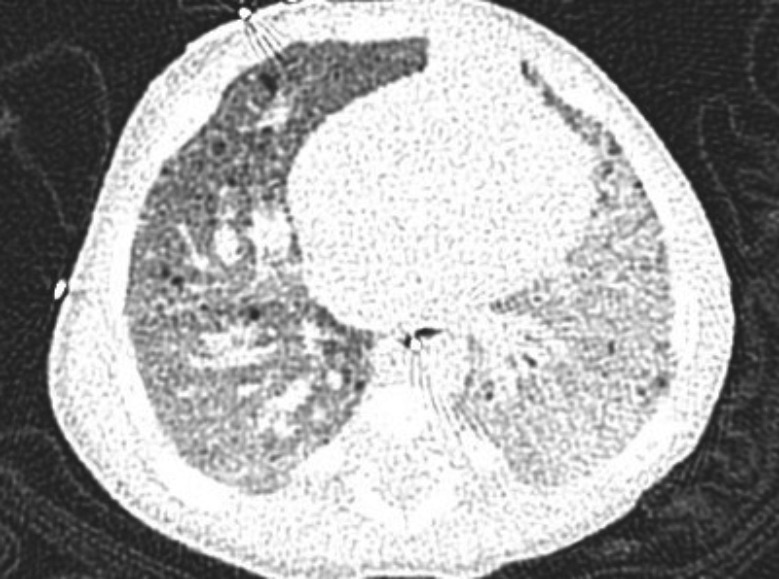
CTA diffuse interstitial reticular markings with multifocal opacities

During his admission, multiple CXR showed persistent bilateral opacities and pulmonary edema as shown in Figure 6. CTA revealed diffuse interstitial reticular markings with multifocal opacities mainly in the upper lobes, and his L PV was not visualized as shown in Figure 7.

V/Q scan showed significant decreased perfusion and ventilation of the L lung (9% and 33%, respectively). Echocardiography showed hypoplastic L PVs.

Since his discharge, he is growing and thriving with no respiratory limitations.

## 4 CASE 3

The middle sister has a history of first‐degree AV block. She is asymptomatic from a respiratory standpoint. Her echocardiogram demonstrated confluent branch pulmonary arteries and a small L PA. Her L PV was not visualized. There were no signs of PAH. Chest CTA demonstrated a hypoplastic left lung and pulmonary artery. Left pulmonary veins were not visualized. Left hemithorax was smaller than the right. Lung ventilation was 23% L, 77% R and perfusion 7% L, 93% R.

Clinical characteristics of the patients are summarized in Table 1.

**Table 1:** Clinical characteristics of the patients

Cases	Gender	Perinatal history	Age at presentation	Additional anomalies	Presenting symptoms	Echocardiogram	Chest CTA	V/Q scan	Lung biopsy	Supportive therapy	Outcome
1	F	Unremarkable	10 y	First‐degree AV block and scoliosis	Cough, chest pain, and hemoptysis	L pulmonary veins were not seen Small L pulmonary artery (LPA) No signs of PH	L pulmonary hypoplasia, L pulmonary veins were not seen near the LA. Patchy ground glass opacities (GGO), interlobular septal thickening, small pulmonary artery, and tiny mediastinal collateral vessels	Global decreased perfusion and ventilation throughout the L lung (6% and 19%, respectively)	L pulmonary veins diminished in size, dilated pleural collaterals, and parenchymal hemorrhage. In addition, consolidation and fibrosis were demonstrated in the LUL and focal segmental cystic lymphangiectasia	L pneumonectomy	At 15 y old, moderate scoliosis (nocturnal brace), restrictive lung function (FVC 47%, stable over 4 y), and intermittent chest pain resolved after 1 y. Mild exercise intolerance
2	M	Preterm 26‐w gestation, high‐risk pregnancy, 43‐y‐old mother, gestational hypertension, cholestasis. C‐section delivery due to fetal distress. Apgar 7, 9	Birth	Bronchopulmonary dysplasia, patent ductus arteriosus, necrotizing enterocolitis, vesicoureteral reflux, congenital glaucoma, increased parenchymal echogenicity of paraventricular white matter on brain US	Respiratory distress at birth	Hypoplastic L pulmonary veins No signs of PH	L pulmonary veins were not seen, diffused interstitial reticular markings with multifocal opacities	Global decreased perfusion and ventilation throughout the L lung (9% and 33%, respectively)	Not done	Continuous positive airway pressure (CPAP) followed by high‐frequency oscillatory ventilation (HFOV) followed by oxygen support	At 3 y, growing, thriving, mild cerebral palsy and developmental delay, and no respirator symptoms. Weaned off oxygen by 12 mo
3	F	Unremarkable	13 y	First‐degree AV block	None	L pulmonary veins were not seen Small LPA No signs of PH	L pulmonary veins were not seen. Hypoplastic L lung. Small pulmonary artery	Global decreased perfusion and ventilation throughout the L lung (7% and 23%, respectively)	Not done	None	Growing, thriving, physically active

## 5 DISCUSSION

This sibling case series demonstrates the variable potential of familial pulmonary vein stenosis. The possibility of autosomal dominance should be considered given that the youngest sibling (case 2) is not paternally related; however, the family declined genetic testing. Given the developmentally normal but small left lung and atretic pulmonary vein in case 1, progressive stenosis rather than atresia is suspected although it cannot be confirmed. The index case has been followed in clinic for 5 years; other than scoliosis that progressed through puberty, treated conservatively with a brace, and restrictive lung disease (stable FVC for 4 years at 47%‐48% predicted) with continued participation in regular physical activity although she was unable to continue competitive running postpneumonectomy. The lymphatic abnormalities noted in her biopsy were attributed to poor venous drainage rather than a primary lymphatic defect, different than at least one other sibling series. Case 2 demonstrates findings consistent with extreme prematurity, including cerebral palsy, as well as the UPVA and V/Q deficits on the affected side. Case 3 is well with no significant clinical history, but with UPVA and hypoplastic L lung is at risk of complications similar to those seen in case 1. Although this condition is rare and these siblings have a heterogeneous presentation, each demonstrates features described in previous cases and case series.

Unilateral pulmonary vein atresia is a rare congenital vascular malformation due to obliteration or absence of the PV on the affected side.^1^ UPVA is a progressive and severe form of pulmonary vein stenosis (PVS). PVS has a prevalence of 1.7 per 100 000 children younger than 2 years of age.^11^ PVS is associated with poor prognosis.^11^

Pulmonary vein stenosis is classified as primary or secondary. Primary (congenital) PVS results from abnormal incorporation of the common PV into the left atrium during embryologic vessel development, leading to partial or complete obliteration of the PV on one or both sides.^10^ Patients usually become symptomatic in the first months to years of life. Approximately 50% of patients with primary PVS have other congenital heart defects (CHD), mostly septal defects.^12^ Primary PVS has also been associated with bronchopulmonary dysplasia (BPD) and preterm birth.^10^, ^11^ In one case study of a consanguineous Turkish family of four siblings with primary PVS, it was associated with antenatally detected lymphatic abnormalities. Gene mapping found the first locus for primary PVS on chromosome 2q35‐2q36.1 (containing 88 genes), suggesting a genetic cause.^16^

Secondary PVS develops due to external compression (such as lymphadenopathy in sarcoidosis, neoplasms, and fibrosing mediastinitis), surgical repair, or cardiac catheterization (most often after correction of total anomalous pulmonary venous drainage in pediatric patients and after ablation or treatment of atrial fibrillation in adults). It can also occur after lung transplantation or lobectomy.^10^

Clinical presentation depends on the number of pulmonary veins involved and the severity of the obstruction.^10^, ^11^ Most patients present in the first months to years of life with cough, exertional dyspnea, respiratory distress, and recurrent pneumonia in the hypoplastic lung (due to impaired mucociliary clearance, local immunity, and venous and lymphatic drainage). As the disease progresses, patients show signs and symptoms of PH, pulmonary edema (due to blockage of lung circulation), and hemoptysis^2^, ^3^ (due to rupture of dilated systemic collateral blood vessels).^10^

Histopathologic changes in the affected lung show hypertrophy and fibrosis of the remaining veins, with intimal fibrosis and reduction of the vascular lumen. Consequently, collateral extrapulmonary vessels develop to partially drain the affected lung. In addition, collaterals between the pulmonary and bronchial circulation that can misinterpreted as pulmonary arteriovenous malformations (PAVM) may develop.^15^ Due to inappropriate gas exchange caused by alterations between ventilation and perfusion, there is a progressive reduction in caliber of the affected PA, which eventually develops flow reversal toward the contralateral artery.^13^ In addition, PH and recurrent infection contribute to the development of parenchymal changes such as ground glass opacities, interlobular septal thickening, and bronchial wall thickening. Pulmonary venous infarction and chronic pulmonary edema can contribute to fibrosis.^5^, ^8^

Unilateral pulmonary vein atresia can be diagnosed through noninvasive tests including echocardiography (PV do not drain into the LA, turbulent flow on Doppler, and monophasic flow or high flow velocities > 1.6 m/s due to significant obstruction), CXR, CTA (detailed PV analysis), and MRI (abnormalities of blood flow in the PA and PV).^10^ Radiographic findings include small ipsilateral hemithorax with mediastinal shift, diminished ipsilateral PA, absent PV drainage into the left atrium, parenchymal abnormalities such as interlobular septal thickening, peribronchovascular thickening, and ground glass opacities. V/Q scan can show reduced perfusion and ventilation to the affected lung.^8^, ^15^ Perfusion deficits may be missed if stenosis is <50%.^15^ Cardiac catheterization was previously the gold standard investigation for PVS; however, with advancements in CTA, invasive catheterization is no longer needed.^5^, ^6^, ^9^

Differential diagnosis includes congenital pulmonary hypoplasia (primary/secondary), poor pulmonary blood flow (Scimitar syndrome and Swyer‐James syndrome), and acquired pulmonary obstruction (mediastinal tumors or pulmonary veno‐occlusive disease).^5^

Treatment options depend on the severity of complications. Asymptomatic patients can be treated conservatively with close follow‐up.^7^ For symptomatic patients, the treatment is surgical. Advances in surgical and interventional catheterization procedures including balloon dilatation and stent insertion are usually unsuccessful. Irreversible changes to the pulmonary parenchyma and vasculature that occur prior to surgery lead to restenosis (up to 50%). Pneumonectomy may be mandatory with uncontrolled hemoptysis,^11^ and rarely, lung transplantation is performed for severe pulmonary hypertension.^10^, ^14^ There are limited data about the role of anticoagulation.^14^ The mortality rate of patients with PVS is high even after surgical repair. Patients with milder degrees of stenosis or only 1 or 2 affected PVs have a better prognosis. Death is usually secondary to pulmonary hypertensive crisis, pulmonary infections, or hemorrhage.^10^

## 6 CONCLUSIONS

Unilateral pulmonary vein atresia is a rare congenital vascular anomaly with obliteration or absence of the pulmonary veins. UPVA carries a significant morbidity and mortality. UPVA can be fatal and should be diagnosed and treated before pulmonary hypertension or massive pulmonary hemorrhage occurs. We present a case series of three siblings with a different paternity and variable presentation of L PVA. Our finding supports the previous Turkish case series suggesting an underlying genetic cause due to the familial presentation (3/3 siblings related through the mother). This sibling trio demonstrated typical features of UPVA including unilateral pulmonary hypoplasia (cases 1, 3); bronchopulmonary dysplasia and preterm birth (case 2); and hemoptysis (case 1). Given the associated morbidity and mortality, this condition should be considered in patients with incidental findings of lung asymmetry. Identifying involved genes will contribute to early diagnosis, future pregnancy planning, and screening of affected families.

## CONFLICT OF INTEREST

Dr Cohn, Dr A Hicks, Dr Lacson, and Dr M Hicks declare that they have no competing interests and did not receive any personal financial support.

## AUTHOR CONTRIBUTIONS

RHEC: conceived and wrote the first draft of the manuscript. AH: identified cases 1 and 3, and revised and reviewed the manuscript. AL: revised and reviewed the manuscript. MH: identified case 2 and suggested the familial linkage, and revised and reviewed the manuscript.
